# Impact of Mode of Delivery on the Survival Rate of Very Low Birth Weight Infants: A Single-Center Experience

**DOI:** 10.7759/cureus.11918

**Published:** 2020-12-05

**Authors:** Mansour A AlQurashi

**Affiliations:** 1 Neonatology Division, Department of Pediatrics, King Abdulaziz Medical City, Ministry of National Guard Health Affairs, Jeddah, SAU; 2 College of Medicine, King Saud Bin Abdulaziz University for Health Sciences, Jeddah, SAU; 3 Research and Development, King Abdullah International Medical Research Center, Jeddah, SAU

**Keywords:** survival rate, very low birth weight, vlbw, cesarean delivery, vaginal delivery

## Abstract

Introduction: Worldwide cesarean birth had increased over the past three decades and in the USA, the overall rate of cesarean birth has increased from 23.8% in 1989 to 31.9% in 2018. Moreover, the substantial increase of preterm infants delivered by cesarean section had reached anywhere from 45% to 72% for gestational age <33 weeks. There is a considerable debate on whether cesarean section confers a survival advantage for preterm infants. Published data on the relationship between mode of delivery and survival rate were inconsistent and there is a lack of large randomized controlled trials (RCTs) that have investigated this important clinical concern. Thus, the aim of this study is to evaluate the impact of cesarean section on the survival rate of very low birth weight (VLBW) infants.

Methods: This was a retrospective cohort study of ≤32 weeks VLBW infants born alive and admitted to Neonatal Intensive Care Unit (NICU) at King Abdulaziz Medical City-Jeddah (KAMC-Jeddah) between January 1, 1994, and December 31, 2019. The primary outcome of interest was the survival rate to discharge of VLBW infants delivered by cesarean section compared to delivered vaginally. Relevant demographic and clinical variables were assessed and its association to survival to discharge of VLBW infants were analyzed.

Results: Of the 1055 ≤32 weeks VLBW infants included in the study, 559 (53%) were delivered by cesarean section, and 496 (47%) were delivered vaginally. Cesarean delivery had increased from 44.2% to 66% between 1994-1998 and 2014-2019, respectively. The rise of cesarean delivery compared with the vaginal delivery was more profound for gestational age ≤26 weeks and birth weight ≤800 g. The VLBW infants delivered by cesarean section had a higher survival rate when compared to infants delivered vaginally (87.29% vs 71.77%, P<0.001). The survival advantage was statistically significant in extremely low birth weight (ELBW) infants (801-1000 g) and infants with birth weight ≤800 g, 86.73% vs 73.62%, P=0.018 and 58.02% vs 40.52, P=0.001, respectively. Moreover, VLBW infants ≤26 weeks gestational age delivered by cesarean section had a higher survival rate of 69.15% vs 44.5%, P<0.001.

Conclusion: This study demonstrates that cesarean birth is associated with higher survival for VLBW infants with birth weight ≤800 g and ELBW infants and gestational age ≤26 weeks compared to vaginal birth.

## Introduction

Worldwide cesarean birth had increased over the past three decades [1] and in the USA, the overall rate of cesarean birth has increased from 23.8% in 1989 to 31.9% in 2018 [2,3]. Moreover, the substantial increase in delivering preterm infants through cesarean section has reached anywhere from 45% to 72% for gestational age <33 weeks [4].

There is a considerable debate on whether cesarean section confers a survival advantage for preterm infants. Published data on the relationship between mode of delivery and survival rate were inconsistent and there is a lack of large randomized controlled trials (RCTs) that have investigated this important clinical concern. Nevertheless, cesarean delivery has been shown to improve the outcome of very low birth weight (VLBW) infants in certain clinical settings like non-vertex fetal presentation and growth-restricted premature infants [5].

There have been two Cochrane reviews that evaluated the role of elective cesarean section versus expectant management and also the impact of cesarean section versus vaginal delivery for preterm birth outcomes; however, these reviews did not find evidence that using cesarean delivery policy would improve outcomes and concluded that the difference between the two groups on mortality was not significant [6,7]. The major limitation of these reviews is that only four trials were included in the final analysis (six trials in total), for a very limited sample of 116 women. Another limitation of these reviews included trials that were rather old and were conducted between 1984 and 1996. Several other cohort studies have found the survival advantage of cesarean birth especially in extremely low birth infants [8-11]. Thus, the aim of this study is to evaluate the impact of cesarean section on the survival rate of VLBW infants.

## Materials and methods

We performed a retrospective cohort study of VLBW infants admitted to Neonatal Intensive Care Unit (NICU) at King Abdulaziz Medical City-Jeddah (KAMC-Jeddah) between January 1, 1994, and December 31, 2019. Perinatal and neonatal data were collected from the unit admission registry and patient medical records were reviewed to ensure completeness and accuracy of the collected data.

The analysis includes VLBW infants who were born alive with gestational age ≤32 weeks. Infants with lethal/complex congenital anomalies or gestational age >32 weeks were excluded from the analysis. The primary outcome of interest was the survival rate to discharge of VLBW infants delivered by cesarean section compared to delivered vaginally. Relevant demographic and clinical variables including the rate of antenatal care, use of antenatal corticosteroid, gestational age (weeks), birth weight (grams), mode of delivery, use of exogenous surfactant, and survival to discharge were collected.

Differences between the two groups were compared using Student’s T-test for the unequal variance of continuous data or Chi-square and Fisher’s exact test for categorical data as appropriate at a threshold P-value of <0.05. The statistical analysis was performed using the Statistical Package for Social Sciences (SPSS), version 23.0 (IBM Corp., Armonk, NY).

## Results

**Figure 1:**
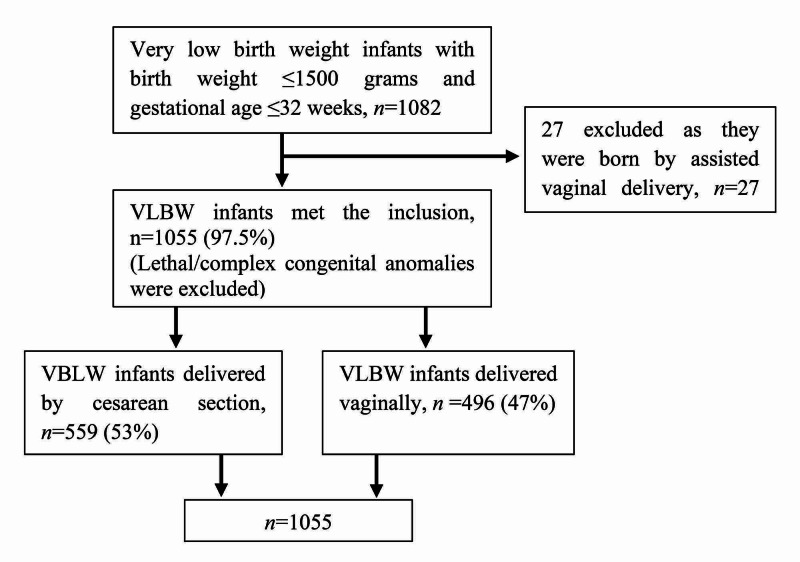
Flow chart of the study eligible cohort population VLBW: very low birth weight.

**Figure 2:**
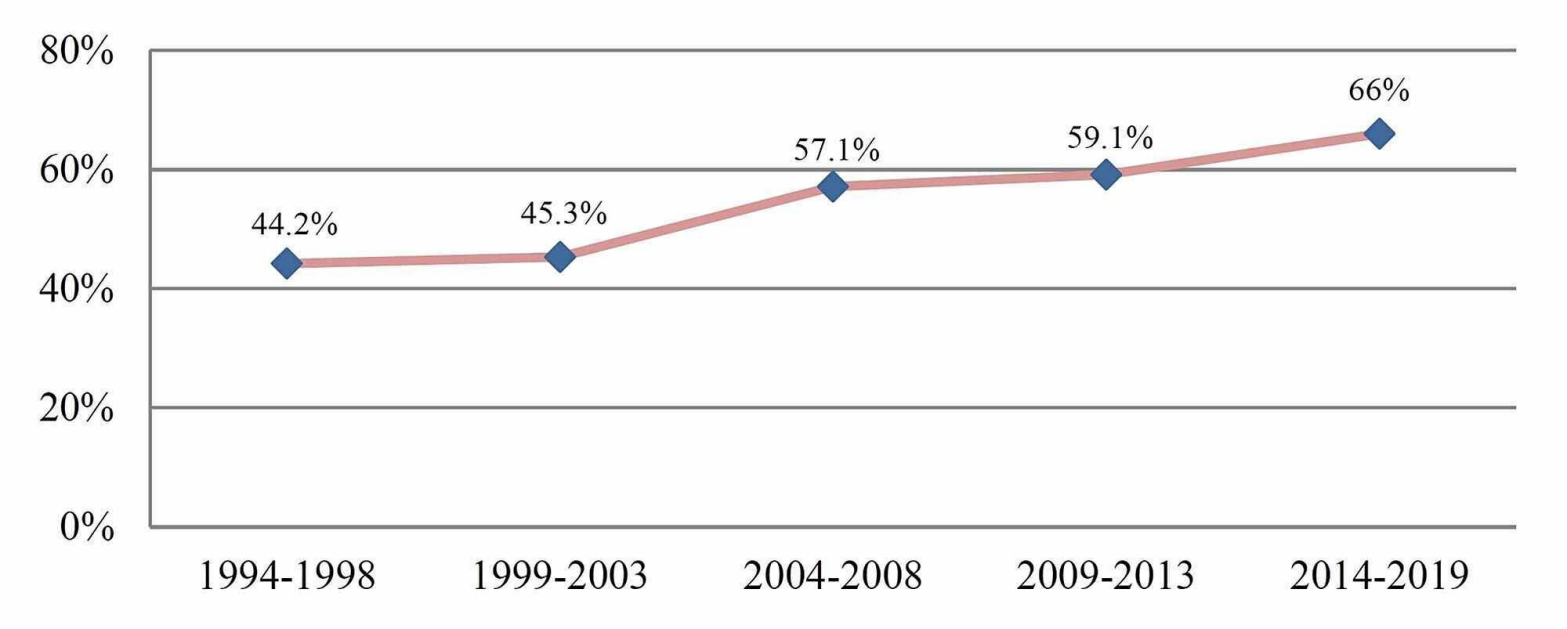
Rate of cesarean delivery among VLBW infants with birth weight ≤1500 g (1994-2019) VLBW: very low birth weight.

**Figure 3:**
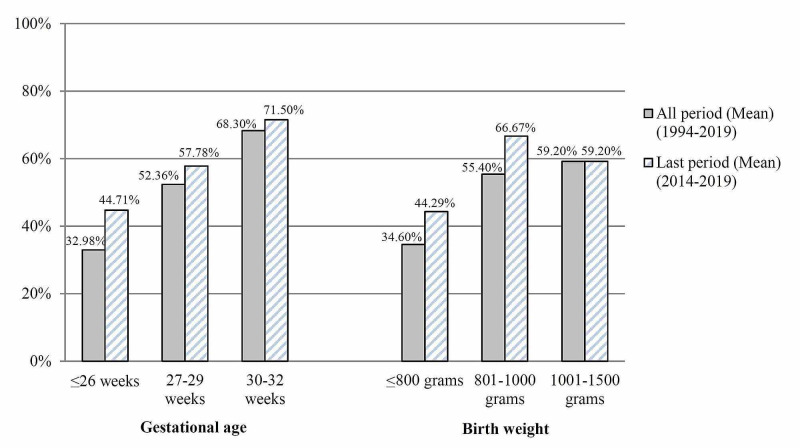
Rate of cesarean delivery among VLBW infants as per gestational age and birth weight categories: cesarean delivery vs vaginal delivery VLBW: very low birth weight.

A total of 1055 VLBW infants were identified and met the study inclusion criteria for analysis (Figure 1). Among them, 559 (53%) were delivered by cesarean section, and 496 (47%) were delivered vaginally. There was a progressive rise in the rate of cesarean delivery for VLBW infants with a gestational age of ≤32 weeks. Cesarean delivery had increased from 44.2% in the years between 1994 and 1998 to reach 66% in the last few years, 2014 and 2019 with 49.3% upward change (Figure 2). The rise of cesarean delivery compared with the vaginal delivery was more profound for gestational age ≤26 weeks and birth weight ≤800 g, 44.71% from 30.65% for gestational age ≤ 26 weeks and 44.29% from 24.14% for birth weights ≤800 g, respectively (Figure 3).

Cesarean section delivery was significantly higher in female compared to male infants, 304/527 (57.7%) vs 223/528 (42.3%), P=0.002. There was no difference between the two groups in the rate of antenatal care, the use of antenatal corticosteroid and surfactant. Infants delivered by caesarian section were of higher gestational age, 29.02 ±2.17 weeks and heavier birth weight (1100±250 g) compared with the gestational age of 27.49 (2.56) weeks and birth weight of 1010±300 g among infants delivered vaginally (Table 1).

**Table 1:** Demographic characteristic of VLBW infants as per the mode of delivery: cesarean delivery vs vaginal delivery VLBW: very low birth weight, NS: not significant, SD: standard deviation.

Variables/characteristics	Total	Cesarean delivery, n (%)	Vaginal delivery, n (%)	Significance
Number	1055	559 (53%)	496 (47%)	--
Gender				
Male	528	255 (48.3%)	273 (51.7%)	0.002
Female	527	304 (57.7%)	223 (42.3%)	
Antenatal care	675 (64%)	370 (66%)	305 (61%)	NS
Antenatal corticosteroid	650 (61.6%)	350 (62.6%)	300 (60.5%)	NS
Surfactant	875 (83%)	462 (82.6%)	415 (83.7%)	NS
Gestational age (weeks) (mean±SD)	28.3 (2.48)	29.02 (2.17)	27.49 (2.56)	<0.001
Birth weight (g) (mean±SD)	1059 (274)	1100 (250)	1010 (300)	<0.001
Birth weight				
≤800 g	234 (22.18%)	81 (34.62%)	153 (65.4%)	<0.001
801-1000 g	204 (19.34%)	113 (55.39%)	91 (44.6%)	
1001-1500 g	617 (58.48%)	365 (59.16%)	252 (40.8%)	
Gestational age				
≤26 weeks	285 (27%)	94 (32.98%)	191 (67.02%)	<0.001
27-29 weeks	382 (36%)	200 (52.36%)	182 (47.64%)	
30-32 weeks	388 (37%)	265 (68.3%)	123 (31.7%)	

**Figure 4:**
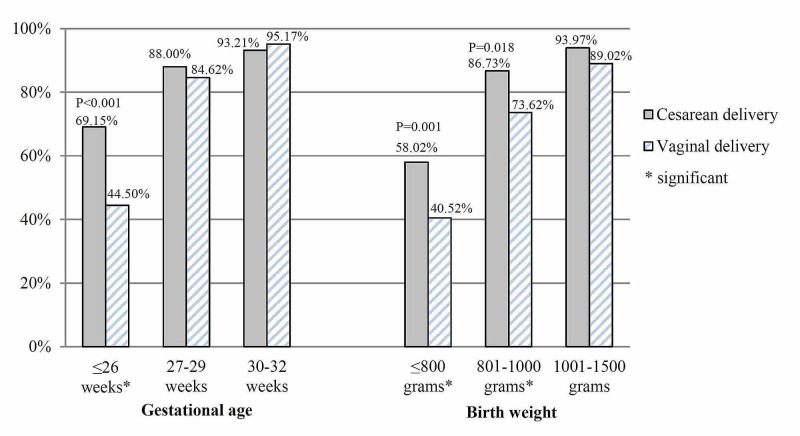
Survival rate of VLBW infants as per gestational age and birth weight categories: cesarean delivery vs vaginal delivery VLBW: very low birth weight.

The VLBW infants delivered by cesarean section had a higher survival rate when compared to infants delivered vaginally (87.29% vs 71.77%, P<0.001). The survival advantage was statistically significant in ELBW infants (801-1000 g) and infants with birth weight ≤800 g, 86.73% vs 73.62%, P=0.018 and 58.02% vs 40.52, P =0.001, respectively. Moreover, VLBW infants of ≤26 weeks gestational age delivered by cesarean section had a higher survival rate of 69.15% vs 44.5%, P<0.001. The survival rate of VLBW was not statistically significant between the two groups for gestational age >26 weeks and birth weight >1000 g (Table 2; Figure 4).

**Table 2:** Survival rate of VLBW infants as per the mode of delivery VLBW: very low birth weight.

Variables/characteristics	Total	Cesarean delivery, n (%)	Vaginal delivery, n (%)	Significance
Number	844/1055 (80%)	488/559 (87.29%)	356/496 (71.77%)	<0.001
Gender				
Male	418/528 (79.17%)	219/255 (85.88%)	199/273 (72.89%)	0.357
Female	426/527 (80.83%)	269/304 (88.49%)	157/233 (67.38%)	
Birth weight				
≤800 g	109/234 (46.58%)	47/81 (58.02%)	62/153 (40.52%)	0.001
801-1000 g	165/204 (80.88%)	98/113 (86.73%)	67/91 (73.62%)	0.018
1001-1500 g	570/617 (92.38%)	343/365 (93.97%)	227/255 (89.02%)	0.073
Gestational age				
≤26 weeks	150/285 (52.63%)	65/94 (69.15%)	85/191 (44.5%)	<0.001
27-29 weeks	330/382 (86.39%)	176/200 (88%)	154/182 (84.62%)	0.335
30-32 weeks	364/388 (93.81%)	247/265 (93.21%)	117/123 (95.12%)	0.466

There was a trend toward higher survival for female VLBW infants particularly with gestational age ≤800 g and gestational age ≤26 weeks (48.3% vs 44.8% for male, P=0.594 and 53.6% vs 51.7%, P=0.755, respectively), but these were not statistically significant. Nevertheless, both male and female VLBW infants delivered by cesarean section had significantly higher survival when compared to infants delivered vaginally if they were evaluated separately (i.e., male and female alone comparing vaginal delivery and cesarean section).

## Discussion

This study found VLBW infants delivered by cesarean section had a higher survival rate than the ones delivered vaginally. The survival advantage was more profound in infants with birth weight ≤800 g and to a lesser extent ELBW infants. There was no difference in survival among the two groups with birth weight between 1001 and 1500 g. Moreover, infants with gestational age ≤26 weeks had a significantly higher survival rate if delivered by cesarean section, however, no difference for gestational age >26 weeks. This has been reported by Holzer et al. wherein appropriate for gestational age infants with gestational age between 23 and 28 weeks had better outcomes after cesarean section delivery (P<0.001 for 23-24 weeks and P=0.001 for 25-28 weeks) [9]. This was also reported by Lee et al. that VLBW infants with vertex presentation and birth weight <1300 g had lower mortality if delivered by cesarean section; however, he recommended further studies to evaluate causality related to the cesarean birth and improved outcomes [12]. The same author had published another study a few months later with an aim to characterize the relationship between cesarean section delivery and death of pre-term vertex neonates and concluded that cesarean section delivery was associated with survival for preterm small-for-gestational-age neonates but not preterm appropriate-for-gestational-age neonates [13]. Deulofeut et al. had reported a trend toward better survival for cesarean birth for infants with birth weight <750 g (65% for cesarean group vs 45% for the vaginal group) but it was not statistically significant [8]. Furthermore, vaginal delivery was a predictor of severe intraventricular hemorrhage (IVH) and poor short-term outcomes [8], and in our study, VLBW infants with birth weight ≤800 g had statistically significant higher survival in the cesarean group (58.02% vs 40.52% with a P-value of 0.001) but we did not report other short-term outcomes apart from rate of survival. Several other cohort retrospective studies did not demonstrate the improved survival based on the method of delivery especially for AGA, preterm infants [14-16]. Wylie et al. had evaluated 2466 vertex preterm VLBW infants and found no survival advantage of cesarean delivery (adjusted OR [95% CI]: 1.08 (0.78-1.49); however, the survival benefit was noted only for growth-restricted infants (adjusted OR [95% CI]: 0.009 (0.02-0.47) [14]. Several RTCs were started to evaluate the impact of mode of delivery on the outcomes of very premature infants, however, they had significant recruitment difficulties and stopped (Personal Communication: MacLennan AH. Randomised Trial of the Mode of Delivery of the Very Premature Infant (Trial Abandoned); 1986) [17-21]. The Cochrane review of these RCTs included only 116 women from six trials (four included in the analysis) with a sample size between 12 and 38. The perinatal death was not significantly different (OR 0.29, 95% CI 0.07-1.14) in three trials. It included 89 women and the conclusion of these reviews is that there is no enough evidence to evaluate the use of the policy of planned immediate cesarean section for preterm infants [6].

Muhuri et al. published an article where he found that among breach/malpresenting preterm infants delivered by primary cesarean section, significantly lower adjusted relative risk for death compared to those delivered vaginally, but vertex presenting neonates results were mixed suggesting decreased relative risk for mortality for those of birth weight <750 g but not significant for 750 - 999 g weight group [22].

Thanh et al. published an article reflecting a secondary analysis of the WHO global survey and Multicountry Survey (MCS) database to investigate the effect of mode of delivery on the outcomes of preterm birth by mode of delivery. The results relevant to our study that compared vaginal birth to cesarean section were associated with significantly decreased fresh stillbirth and perinatal deaths in preterm birth with gestational age between 22 and 37 weeks but associated with higher maternal and neonatal ICU admissions. Hence, the justification for the preferred mode of delivery should be carefully considered when deciding the mode of delivery for preterm infants [23].

Prospective RCTs are the scientific method to alleviate the dilemma of the cesarean section delivery benefit vs vaginal birth for preterm infants’ debate; however, such trials are not currently feasible in order to reach a solid conclusion particularly for vertex presenting preterm fetuses. The hypothetical advantages for cesarean birth vs vaginal route are to avoid prolonged labor, and possibly less traumatic is the suggested benefit factor; nevertheless, this controversial topic among the obstetric and neonatal community was debated over the past two decades but clear and solid recommendations are difficult to reach yet.

There are several limitations to our study. For its retrospective nature, some perinatal variables of importance were not included. The data neither distinguish between emergency and elective cesarean birth nor did include information on the specific indications for performing cesarean section or fetal presentation whether it was vertex or non-vertex, and all these variables are important when the method of delivery is considered.

## Conclusions

This study demonstrates that cesarean birth is associated with higher survival for VLBW infants with birth weight ≤800 g and ELBW infants and gestational age ≤26 weeks compared to vaginal birth. VLBW infants of gestational age >26 weeks and birth weight more than 1000 g had no difference in survival rate between the two groups. Further studies are needed to further characterize the important factors and clinical variables that may affect the outcome of VLBW infants based on the method of delivery.

## References

[1] Betrán AP, Ye J, Moller AB, Zhang J, Gülmezoglu AM, Torloni MR (2016). The increasing trend in caesarean section rates: global, regional and national estimates: 1990-2014. PLoS One.

[2] Taffel SM, Placek PJ, Moien M, Kosary CL (1991). 1989 US cesarean section rate steadies - VBAC rate rises to nearly one in five. Birth.

[3] Martin JA, Hamilton BE, Osterman MJK, Driscoll AK (2019). Births: final data for 2018. Natl Vital Stat Rep.

[4] Stohl HE, Szymanski LM, Althaus J (2011). Vaginal breech delivery in very low birth weight (VLBW) neonates: experience of a single center. J Perinat Med.

[5] Lodha A, Zhu Q, Lee SK, Shah PS, Canadian Neonatal Network (2011). Neonatal outcomes of preterm infants in breech presentation according to mode of birth in Canadian NICUs. Postgrad Med J.

[6] Alfirevic Z, Milan SJ, Livio S (2013). Caesarean section versus vaginal delivery for preterm birth in singletons. Cochrane Database Syst Rev.

[7] Grant A, Glazener CM (2001). Elective caesarean section versus expectant management for delivery of the small baby. Cochrane Database Syst Rev.

[8] Deulofeut R, Sola A, Lee B, Buchter S, Rahman M, Rogido M (2005). The impact of vaginal delivery in premature infants weighing less than 1,251 grams. Obstet Gynecol.

[9] Holzer I, Lehner R, Ristl R, Husslein PW, Berger A, Farr A (2017). Effect of delivery mode on neonatal outcome among preterm infants: an observational study. Wien Klin Wochenschr.

[10] Malloy MH (2008). Impact of cesarean section on neonatal mortality rates among very preterm infants in the United States, 2000-2003. Pediatrics.

[11] Jonas HA, Khalid N, Schwartz SM (1999). The relationship between Caesarean section and neonatal mortality in very-low-birthweight infants born in Washington State, USA. Paediatr Perinat Epidemiol.

[12] Lee HC, Gould JB (2006). Survival advantage associated with cesarean delivery in very low birth weight vertex neonates. Obstet Gynecol.

[13] Lee HC, Gould JB (2006). Survival rates and mode of delivery for vertex preterm neonates according to small- or appropriate-for-gestational-age status. Pediatrics.

[14] Wylie BJ, Davidson LL, Batra M, Reed SD (2008). Method of delivery and neonatal outcome in very low-birthweight vertex-presenting fetuses. Am J Obstet Gynecol.

[15] Zhu JJ, Bao YY, Zhang GL, Ma LX, Wu MY (2014). No relationship between mode of delivery and neonatal mortality and neurodevelopment in very low birth weight infants aged two years. World J Pediatr.

[16] Werner EF, Savitz DA, Janevic TM, Ehsanipoor RM, Thung SF, Funai EF, Lipkind HS (2012). Mode of delivery and neonatal outcomes in preterm, small-for-gestational-age newborns. Obstet Gynecol.

[17] Zlatnik FJ (1993). The Iowa premature breech trial. Am J Perinatol.

[18] Penn ZJ, Steer PJ (1990). Reasons for declining participation in a prospective randomized trial to determine the optimum mode of delivery of the preterm breech. Control Clin Trials.

[19] Viegas OA, Ingemarsson I, Sim LP (1985). Collaborative study on preterm breeches: vaginal delivery versus caesarean section. Asia Oceania J Obstet Gynaecol.

[20] Lumley J, Lester A, Renou P, Wood C (1985). A failed RCT to determine the best method of delivery for very low birth weight infants. Control Clin Trials.

[21] Wallace RL, Schifrin BS, Paul RH (1984). The delivery route for very-low-birth-weight infants. A preliminary report of a randomized, prospective study. J Reprod Med.

[22] Muhuri PK, Macdorman MF, Menacker F (2006). Method of delivery and neonatal mortality among very low birth weight infants in the United States. Matern Child Health J.

[23] Thanh BYL, Lumbiganon P, Pattanittum P (2019). Mode of delivery and pregnancy outcomes in preterm birth: a secondary analysis of the WHO Global and multi-country Surveys. Sci Rep.

